# Spatially Adaptive Regularization in Total Field Inversion for Quantitative Susceptibility Mapping

**DOI:** 10.1016/j.isci.2020.101553

**Published:** 2020-09-12

**Authors:** Priya S. Balasubramanian, Pascal Spincemaille, Lingfei Guo, Weiyuan Huang, Ilhami Kovanlikaya, Yi Wang

**Affiliations:** 1 Electrical and Computer Engineering, Cornell University, Ithaca, NY 14853, USA; 2 Department of Radiology, Weill Cornell Medicine, New York, NY 10065, USA; 3 Biomedical Engineering, Cornell University, Ithaca, NY 14853, USA

**Keywords:** Nuclear Magnetic Resonance, Magnetism, Physics Magnetic Resonance Imaging, Algorithms

## Abstract

Adaptive Total Field Inversion is described for quantitative susceptibility mapping (QSM) reconstruction from total field data through a spatially adaptive suppression of shadow artifacts through spatially adaptive regularization. The regularization for shadow suppression consists of penalizing low-frequency components of susceptibility in regions of small susceptibility contrasts as estimated by R2∗ derived signal intensity. Compared with a conventional local field method and two previously proposed regularized total field inversion methods, improvements were demonstrated in phantoms and subjects without and with hemorrhages. This algorithm, named TFIR, demonstrates the lowest error in numerical and gadolinium phantom datasets. In COSMOS data, TFIR performs well in matching ground truth in high-susceptibility regions. For patient data, TFIR comes close to meeting the quality of the reference local field method and outperforms other total field techniques in both clinical scores and shadow reduction.

## Highlights

• TFIR's adaptive regularization obtains magnetic susceptibility from magnetic field
• TFIR has low artifact incidence on both quantitative and clinical scores
• The error for TFIR is low on various numerical and ground truth tests
• Clinical applications for TFIR include hemorrhages and whole head mapping

## Graphical Abstract

**Figure:**
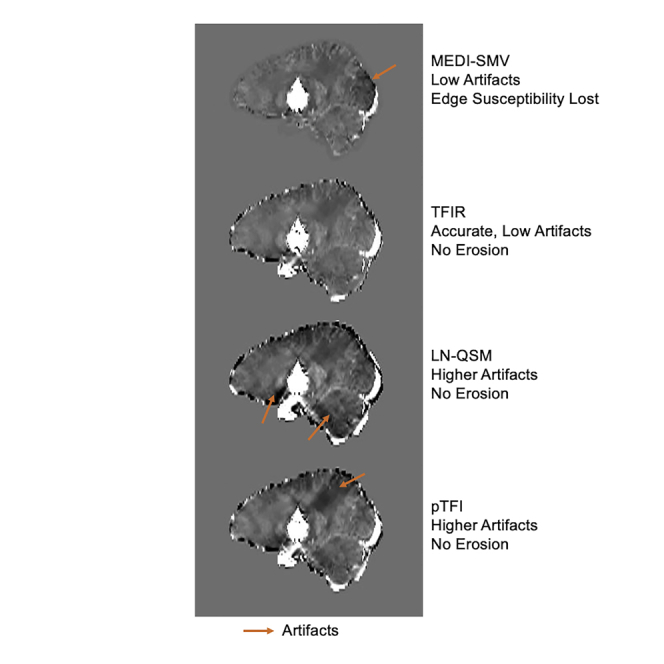

## Introduction

Quantitative susceptibility mapping (QSM) aims to solve the inverse problem of mapping the magnetic susceptibility from the measured magnetic field. QSM applications include targeting for deep brain stimulation (Liu et al., 2013a), monitoring multiple sclerosis (Li et al., 2015), distinguishing calcification and hemorrhage (Deistung et al., 2013), dating and monitoring cerebral cavernous malformations (Tan et al., 2014), Alzheimer's disease (Acosta-Cabronero et al., 2013), Parkinson's disease (Murakami et al., 2015), mapping of magnetic nanocarrier distribution (Kirui et al., 2013; Liu et al., 2010), and liver iron content measurements (Li et al., 2018).

Current QSM methods typically perform brain extraction that may be followed by additional erosion, either as part of the background field removal process (Schweser et al., 2011) or due to the inclusion of a spherical mean value (SMV) operator in the dipole inversion process (Wang and Liu, 2015), referred to in the following as Morphology Enabled Dipole Inversion with SMV or MEDI-PDF-SMV, shortened to MEDI-SMV for the remainder of this paper. This type of local field method has been utilized prior to the introduction of total field methods and, with the introduction of the SMV operator, is successful at suppressing shadow artifacts, as described in the literature (Kee et al., 2017). It is of interest to map the susceptibility of the entire brain. Methods have been proposed to compute a susceptibility map directly from the total field to avoid the propagation of background removal errors into the final susceptibility map. The susceptibility map to be computed in these methods has a large dynamic range: brain tissue susceptibility falls roughly within the −0.1- to 0.3-ppm range, the susceptibilities of bone (−2 ppm) and the air (9 ppm) are one or two orders of magnitude larger. A straightforward dipole field inversion of the resulting tissue field often leads to large residual streaking and shadow artifacts (Li et al., 2015; Shmueli et al., 2009).

In recent years, a number of total field methods have been proposed (Chatnuntawech et al., 2017; Liu et al., 2013b, 2017; Sharma et al., 2005; Sun et al., 2018; Wang and Liu, 2015). In Least Norm QSM (LN-QSM), L2 regularization is used to fit the total field and reconstruct a susceptibility map (Sun et al., 2018). In Liu et al., 2017, preconditioning is used based on the expected covariance of the solution: the preconditioner *P* for which *P*^*H*^*P* is approximately equal to covariance matrix Γ of the solution. The resulting method (preconditioned TFI or pTFI) has been shown to provide an accelerated algorithm convergence and reduce streaking and shadow artifacts without mask erosion. There are two published methods that propose preconditioned total field inversion (Liu et al., 2017) (Liu et al., 2020). Both these methods use R2∗ or initial susceptibility estimates to estimate the covariance matrix and use these as preconditioners. It should be noted that preconditioned total field inversion in both adaptive and binary variants of preconditioner published (Liu et al., 2017, 2020) have a similar rationale mathematically, although the implementation and resultant artifact incidence is different. In Chatnuntawech et al., a single-step QSM method is proposed that does not require separate background field removal and is shown to have a lower error than competing local field algorithms. The leading question in the development of new algorithms is matching the quality of the soft tissue region of the brain using artifact suppressing local field methods such as MEDI-SMV with the image quality of total field methods in addition to obtaining highly accurate mapping of typically eroded head regions (such as the sinus, skull, and scalp).

This paper introduces a regularized total field inversion (TFIR) method that operates on the principles of spatially adaptive regularization, building off of the recent development by Sun et al. (2018). The regularization operator is a low-pass filter that is spatially weighted based on the R2∗ map obtained from the same gradient echo data. This regularization is designed to suppress low spatial frequency components in the susceptibility solution that are not present in the R2∗ map. For all data considered in this study, TFIR was compared with MEDI-SMV (nonlinear signal model) (Liu et al., 2013b), pTFI (Liu et al., 2017), and LN-QSM (Sun et al., 2018) in phantom, healthy subjects, and patients, both with and without hemorrhage.

### Proposed Technique and Mathematical Rationale

In tissue magnetism, both the background susceptibility sources *χ*_*b*_ (the sources outside the region of interest and that give rise to the background field *F*_*b*_) and the local susceptibility sources *χ*_*l*_ (defined as the sources inside the region of interest and that give rise to the local field *F*_*l*_) contribute to the observed total field *f*:

$$f=F_{l}+F_{b}=d*\chi =d*\left(\chi_{l}+\chi_{b}\right)$$

In conventional local field inversion, a spherical mean value (SMV) operator is included in the kernel in the dipole inversion step in order to suppress residual background fields in the estimated local field. The original optimization problem is given by

Equation (1) $$\chi^{*}=\underset{\chi }{\text{argmin}}\frac{1}{2}||w\left(f-d*\chi \right)|{|}_{2}^{2}+\lambda_{1}||M_{G}\nabla \chi |{|}_{1}$$

In conventional local field inversion, error may occur in the background field removal, which will lead to errors in the estimated local susceptibility appearing at shadowing artifacts in QSM. A spherical mean value (SMV) operator may be included in the kernel in the dipole inversion step or a total field inversion may be used to suppress residual background fields in the estimated local field. Additionally, Equation 1 ignores possible tissue anisotropy and suffers from digitization error when fitting imaging data, which also contribute substantially to shadowing artifacts in QSM (Kee et al., 2017).

Shadowing artifacts can be reduced using a regularization that imposes uniform susceptibility in regions known to be uniform, such as ventricles with cerebrospinal fluid in the brain, as in MEDI+0 (Liu et al., 2018). There are still residual shadowing artifacts obvious in regions with low susceptibility contrasts. To address this problem, we propose to generalize this regularization using spatial adaptation. The QSM reconstruction problem in the total field inversion framework may be formulated specifically as:

Equation (2) $$\chi^{*}=\underset{\chi }{\text{argmin}}\frac{1}{2}||w\left(f-d*\chi \right)|{|}_{2}^{2}+\lambda_{1}||M_{G}\nabla \chi |{|}_{1}+\lambda_{2}||rL\chi |{|}_{2}^{2}$$

The third regularization term provides spatially adaptive information, where *L* is a low-pass filter, *λ*_2_ a regularization parameter, and *r* a weighting mask derived below by using R2∗ information to obtain signal intensity, which is used as the adaptive information. This effectively penalizes susceptibility variation heavily in regions with expected uniformity or low R2∗, moderately in regions with moderate susceptibility contrasts or moderate R2∗, and minimally in regions with large susceptibility contrasts or large R2∗. In this work, *L* was chosen to be the spherical mean value operator with radius *k*, whereas the weighting mask *r* was set to:

$$r=e^{-\left|\tau LR_{2}^{*}\right|}\text{,}$$

where *τ* is a constant parameter (in seconds) and the exponential function scales the penalty from uniform to high-contrast regions. Equation 2 may also be regarded as a generalization of the recently developed LN-QSM technique, by applying spatial adaptation on the L2 norm regularization for the benefit of shadow artifacts suppression.

**Figure 1:**
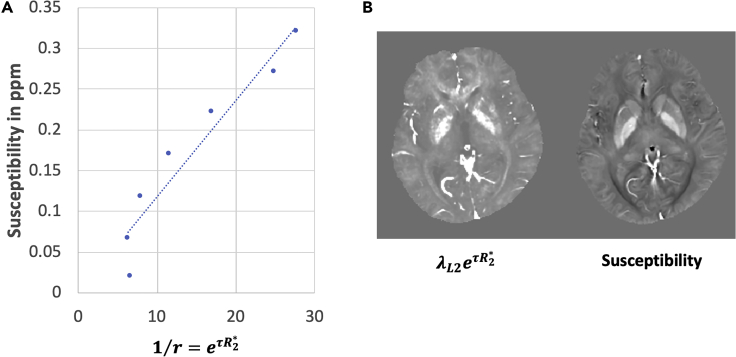
Rationale for TFIR Regularization (A and B) (A) Correlation between susceptibility and spatially adaptive masking term. (B) Comparison between the point-wise inverse of the weighting mask *r* and susceptibility map in a healthy subject. Here, 1/*r* = *e*^*tR*^_2_^∗^ and the displayed imaged is scaled by *λ*_*L*2_ to maintain the window level.

The variable weighting mask r provides spatially adaptive regularization, which allows artifact suppression and accurate solutions of inverse problems (Li, 2011; Song et al., 2015). In order to derive a proper weighting factor for spatially adaptive regularization, it is important to obtain information regarding the spatial content of the image. Research on preconditioners and regularization terms provide backing for choosing adaptive weighting factors that resemble the expected solution (Kee et al., 2017; Liu et al., 2017, 2020). Thus, the motivation for the above chosen weighting factor preceding the L2 regularization in TFIR is the observation that the point-wise inverse of the signal magnitude at later echo times (over 50 ms) resembles the susceptibility map. Figure 1A shows the correlation between the median susceptibility and 1/*r* values of pixels binned according to their susceptibility. For this figure, susceptibility values were binned into seven regions each covering a range of 0.05 ppm from [0, 0.35] ppm in order to reduce noise fluctuations and outliers. Then, the median R2∗ for each of these bins was computed and a regression was computed between resulting susceptibility and 1/*r* values. This is also visually illustrated in Figure 1B, where a susceptibility map is compared with the point-wise inverse of the spatially adaptive regularization mask, demonstrating resemblance. Appropriate values for the regularization parameter *λ*_2_, time constant *τ*, and radius *k* of the low pass filter *L* are empirically chosen by minimizing reconstruction error in a representative subject for which a ground truth susceptibility map was available. In this work, this was given by a COSMOS reconstruction, which required the acquisition of data in multiple orientations with respect to B_0_ (Liu et al., 2009). The resulting technique will be referred to here as TFIR (Adaptive Total Field Inversion Regularized). It should be noted that LN-QSM also utilizes a spatially adaptive technique in the sense that a binary mask corresponding to brain tissue is used in the L2 regularization. TFIR takes this methodology a step further by incorporating tissue-specific, spatially adaptive information through variable masking.

## Results

### Parameter Optimization

**Figure 2:**
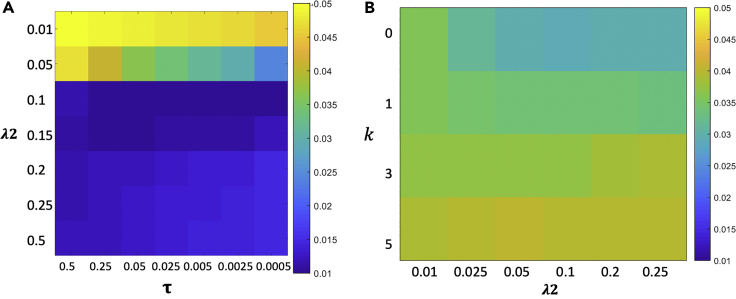
Regularization Parameter Optimization (A) Optimal regularization parameters using COSMOS error minimization shown for *τ*, *λ*_2_. (B) Optimal regularization parameters using COSMOS error minimization shown for *k*, *λ*_2_. Colormap units for (A) and (B) in ppm.

The reconstruction error for the TFIR method compared with the COSMOS ground truth for one subject is depicted in Figure 2. Minimum error was found for *λ*_2_ = 0.1 and *τ* = 0.05*s* and a *k* = 1 *mm* radius for *L*. These values were used for all remaining reconstructions in this paper. Given the COSMOS evaluation performed for the optimal kernel size selection, all subjects are analyzed with a 1-mm kernel.

### Gadolinium Phantom

**Figure 3:**
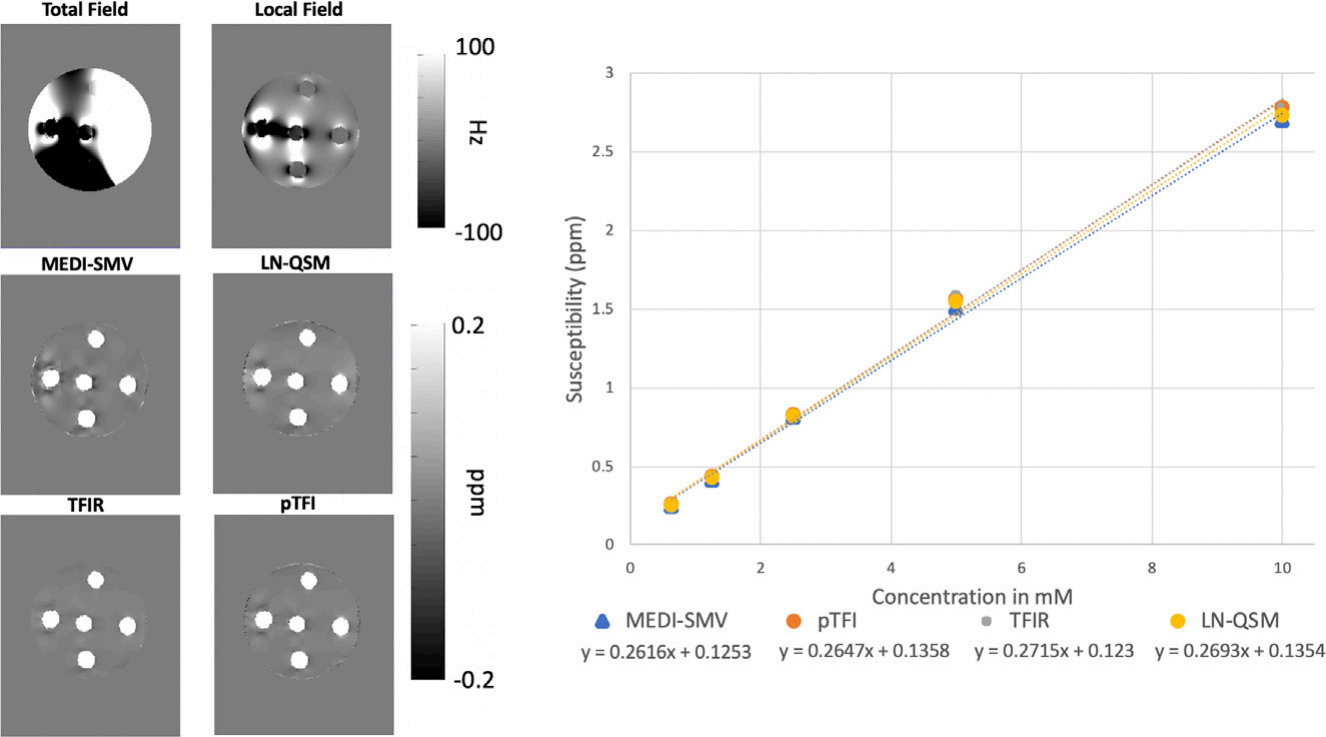
Gadolinium Phantom Analysis Comparison of MEDI-SMV, LN-QSM, pTFI, and TFIR in the Gadolinium phantom (left). The corresponding linear regression of the measured Gd concentrations (right). Ground truth fit should have a slope of 0.33 ppm/mM.

A comparison of MEDI-SMV, LN-QSM, pTFI, and TFIR in the gadolinium phantom is shown in Figure 3. As described in the methods section, the gadolinium phantom background field was increased in strength by including a numerically simulated external susceptibility source to make the results more relevant to the process of total field reconstruction. Reconstruction parameters were *λ*_1_ = 0.01, *λ*_2_ = 0.1 and *τ* = 0.05 s and *k* = 1 mm for TFIR, p = 100 and *λ*_1_ = 0.01 for pTFI, *λ*_1_ = 5 x 10^−4^ and *λ*_2_ = 1 x 10^−3^ for LN-QSM, and *λ*_1_ = 0.01 For MEDI-SMV. All methods use an edge mask that preserves 10% of the edge voxels found through optimization. Shadowing artifacts within the agarose are reduced for TFIR compared with LN-QSM and pTFI and are comparable with those of MEDI-SMV (Figure 3). Linear regression between the reconstructed and measured susceptibilities (Figure 3) showed a slope of 0.262, 0.269, 0.265, and 0.271 ppm/mM for MEDI-SMV, LN-QSM, pTFI, and TFIR, respectively, with a true slope of 0.33. Total field reconstructions are performed with the total field and no background removal. The total field and the local field are both shown in Figure 3 at the top.

### Numerical Phantom

**Figure 4:**
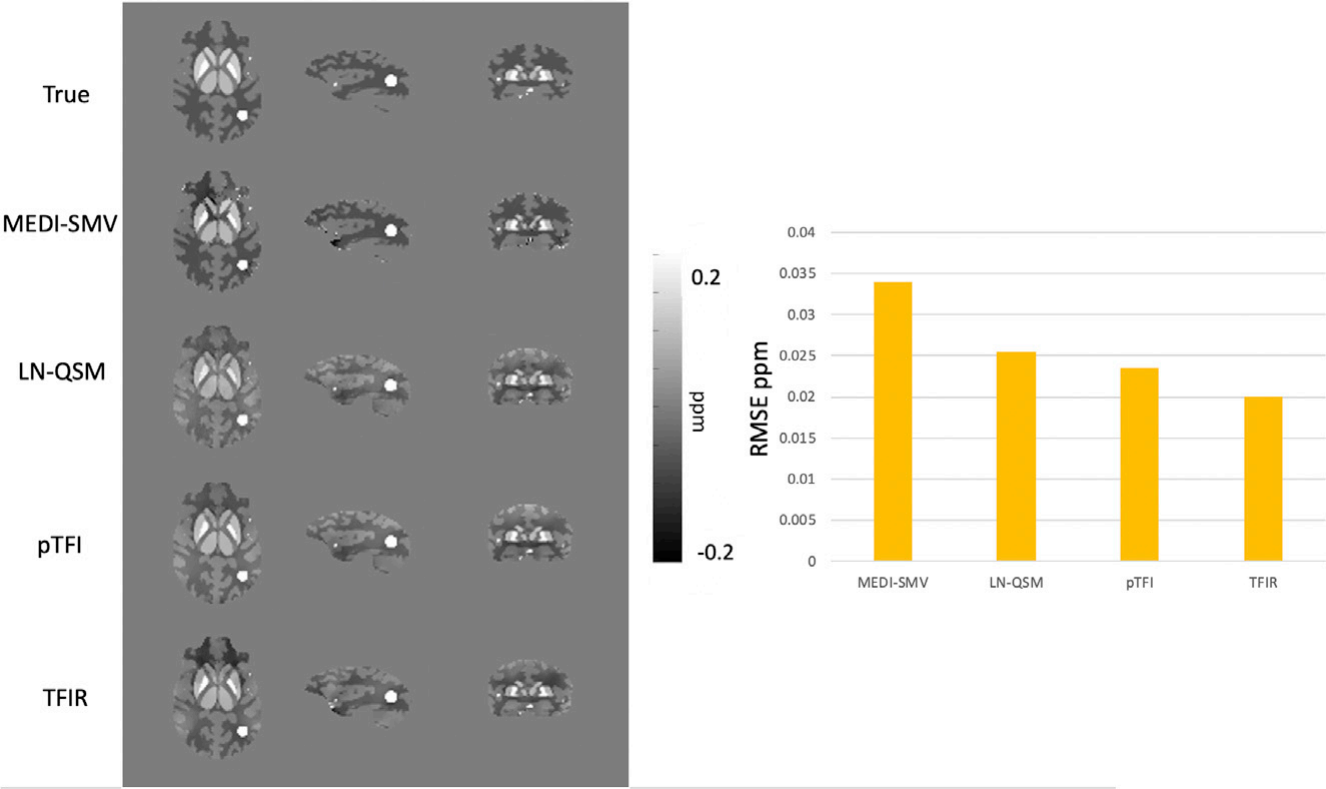
Numerical Phantom Analysis Comparison of MEDI-SMV, LN-QSM, pTFI, and TFIR in the numerical brain simulation (left) and corresponding RMSE for each method (right).

A comparison of the ground truth and the MEDI-SMV, LN-QSM, pTFI, and TFIR reconstructions for the numerical phantom is shown in Figure 4. For TFIR, parameters were *λ*_1_ = 1∙10^−3^, *λ*_2_ = 0.025, *τ* = 0.05 s, and the *k* = 1 mm. Parameters for MEDI-SMV were *λ*_1_ = 2∙10^−2^ and 5 mm SMV. Parameters for pTFI were p = 200 and *λ*_1_ = 2∙10^−3^. Parameters for LN-QSM were *λ*_1_ = 1.25∙10^−3^ and *λ*_2_ = 3.75 x 10^−3^ with magnitude masking to produce the M term in the governing equation. For this section, LN-QSM was modified with modifications suggested in the original work in the whole head reconstruction section. Details are in equation T2 of the Transparent Methods section. The phantom in this simulation has a substantial background field induced by setting the background susceptibility to 9 ppm to mimic air. The corresponding RMSE values are shown in Figure 4. All methods result in excellent accuracy with TFIR producing the lowest error of 0.02 ppm RMSE.

### Healthy Subjects

**Figure 5:**
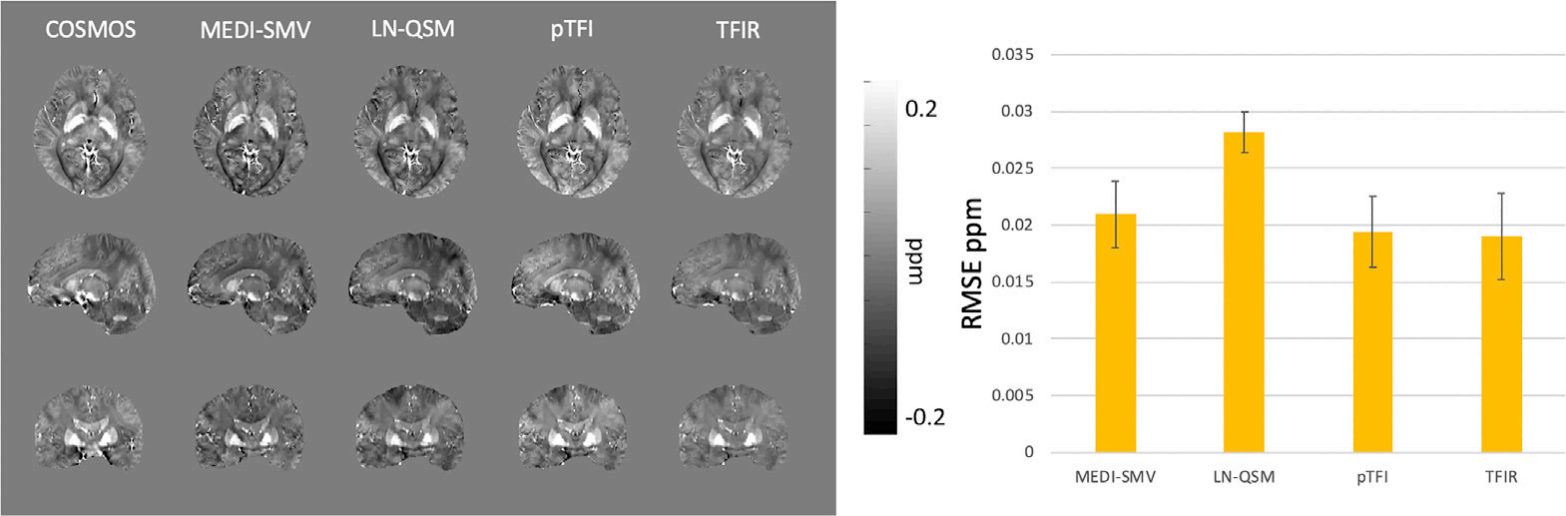
COSMOS Ground Truth Analysis Comparison of COSMOS, TFIR, LN-QSM, and MEDI-SMV in one healthy subject in axial, sagittal, and coronal orientations (left) and the corresponding RMSE across four subjects (right).

Reconstruction parameters were as follows. *λ*_1_ was set to 1∙10^−3^ for all methods. SMV kernel was set to 5 for MEDI-SMV. λ_2_ = 2.5∙10^−3^ for LN-QSM, p = 30 for pTFI, and λ_2_ = 0.1, τ = 0.05 s, and the k = 1 mm for TFIR. Figure 5 shows a comparison between TFIR, LN-QSM, pTFI, and MEDI-SMV and COSMOS in axial, sagittal, and coronal orientation in one healthy subject. TFIR provided the lowest RMSE compared with the other competing methods (Figure 5).

**Figure 6:**
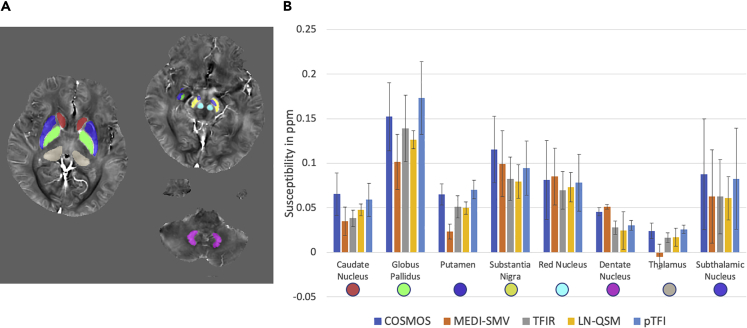
ROI Analysis across Four Healthy Subjects for which a COSMOS Reconstruction Is Available (A–C) (A) COSMOS reconstruction showing the various ROIs. (B) Comparison of ROI values across all methods, (C) percent errors for each ROI and method.

Results from the ROI analysis are shown in Figure 6. None of the presented methods present clear advantages or disadvantages. MEDI-SMV underestimated the values of the Caudate Nucleus, Globus Pallidus, Putamen, and Thalamus. The proposed method had a lower error than MEDI-SMV for the Caudate Nucleus, Globus Pallidus, Putamen, Subthalamic Nucleus, Thalamus, and Red Nucleus and higher error than MEDI-SMV for the Substantia Nigra and Dentate Nucleus.

### Clinical Data

**Figure 7:**
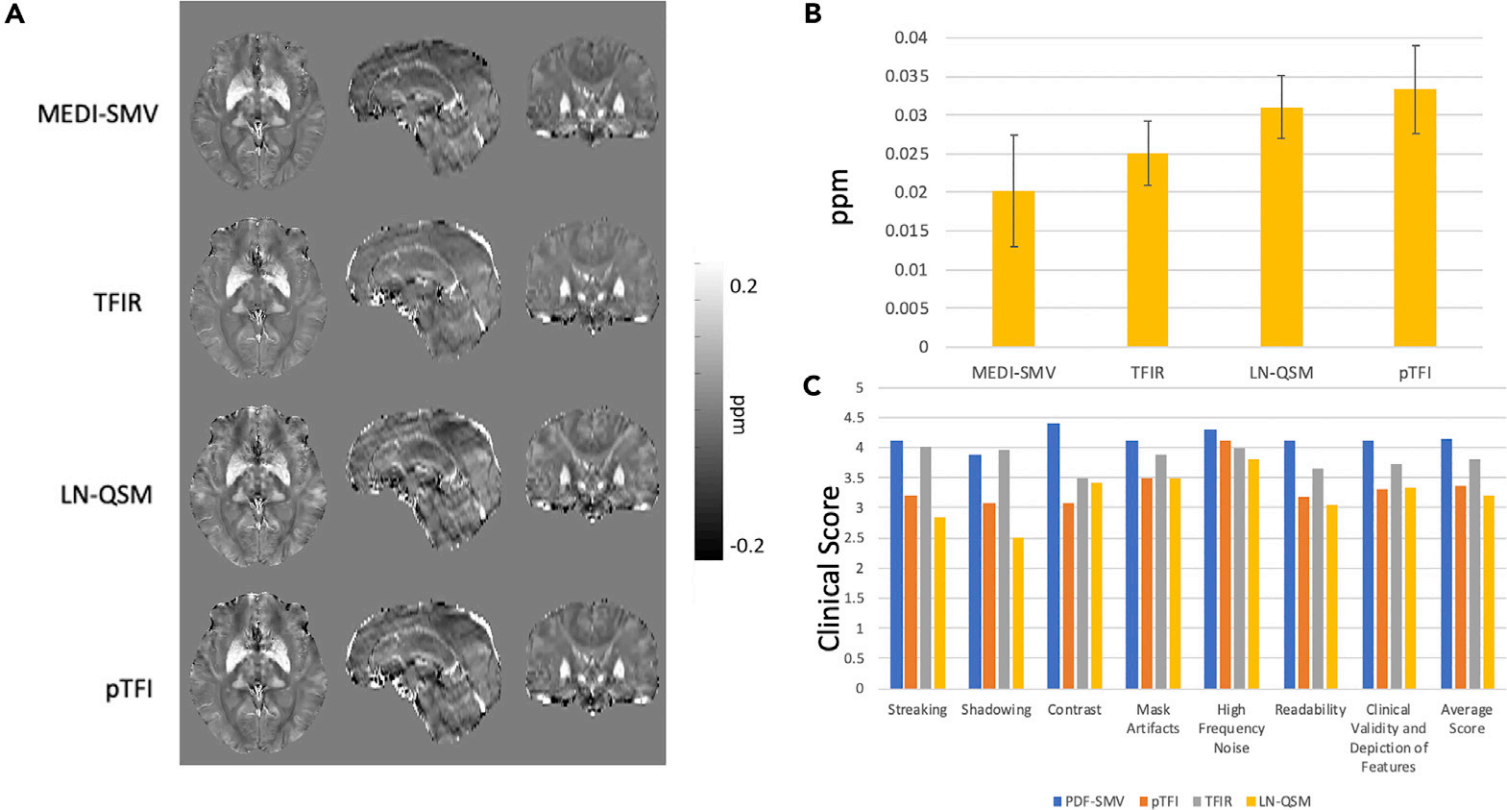
Nonhemorrhage Case Analysis (A–C) (A) Comparison of MEDI-SMV, LN-QSM, TFIR, and pTFI in a non-hemorrhage containing dataset with (B) shadow index across 16 subjects and (C) clinical score. All reconstructions are shown in the same mask as used by MEDI-SMV. See also Figure S2.

**Figure 8:**
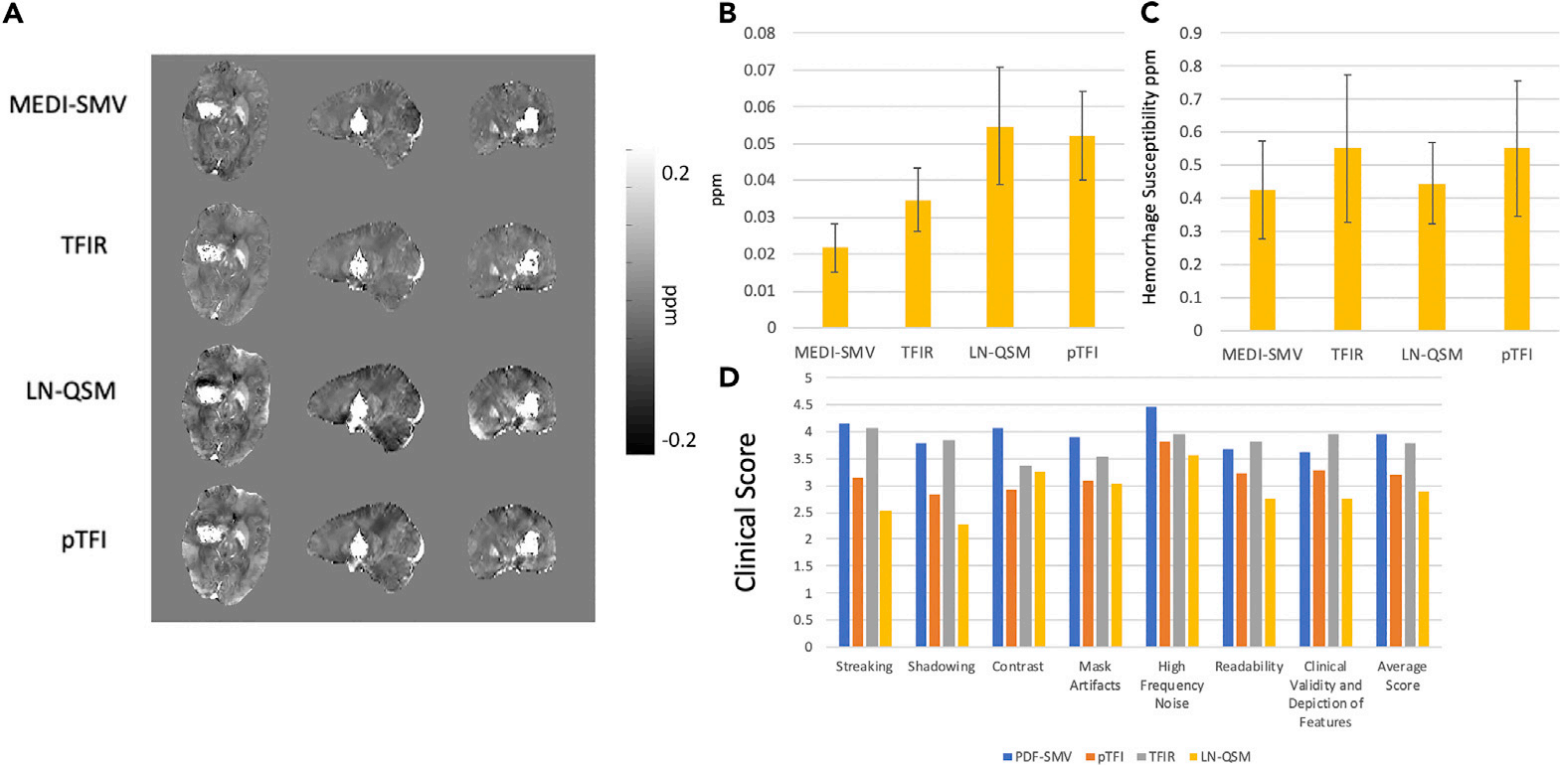
Hemorrhage Case Analysis (A–D) (A) Visual depiction of MEDI-SMV, LN-QSM, pTFI, and TFIR in a hemorrhage containing dataset with (B) shadow index across 17 subjects, (C) average hemorrhage susceptibility across 17 subjects, and (D) clinical scores across 17 subjects. All reconstructions are shown in the same mask as used by MEDI-SMV. See also Figure S3.

Reconstruction parameters follow closely with the healthy data optimization from the preceding section. *λ*_1_ was set to 1∙10^−3^ for all methods. SMV kernel was set to 5 for MEDI-SMV. *λ*_2_ = 2.5∙10^−3^ for LN-QSM, p = 30 for pTFI, and *λ*_2_ = 0.1, *τ* = 0.05s, and the *L* kernel radius 1 mm for TFIR. Modifications made to the LN-QSM method, similar to those in the phantom experiments, are detailed in equation T2 of the Transparent Methods section. Among 33 patients, 16 were found to have no hemorrhage. Clinical scores provided by three experienced radiologist and a quantitative shadow index (described in the Transparent Methods, equation T1) were obtained for all 33 datasets. Visual comparisons and quantitative measures can be found for both non-hemorrhage and hemorrhage cases in Figures 7 and 8, respectively. Among the non-hemorrhage patients, the shadow index was 20.2, 25.1, 31.0, 33.3 ppb for MEDI-SMV, TFIR, LN-QSM, and pTFI, respectively. In Figure 7, among the total field methods, TFIR showed the lowest amount of shadow artifact and came close in apparent image quality to MEDI-SMV. In Figure 8, reconstructions and results are shown for hemorrhage cases. Among the total field methods, LN-QSM and pTFI showed shadowing and streaking on a similar level. TFIR provided the most improvement for the total field reconstructions of hemorrhages in shadow and streaking artifacts, as illustrated by the better shadow index and clinical scores. This is also visible in the example images displayed in Figures 7 and 8. The shadow index averaged over all hemorrhage patients was 21.8, 34.5, 54.7, 52.1 ppb for MEDI-SMV, TFIR, LN-QSM, and pTFI, respectively, again with TFIR producing the lowest shadowing out of the total field methods tested as seen in Figure 8. Figure S1 provides an illustration of how the shadow index is computed for two examples: a low and a high artifact reconstruction. For pTFI, LN-QSM, and TFIR, reconstructions using the original mask for Figures 7 and 8 are shown in Figures S2 and S3, respectively.

Clinical scores (with higher indicating better image quality) are shown in Figures 7 and 8 for the set of non-hemorrhage and hemorrhage cases, respectively. Average clinical scores across the 16 non-hemorrhage subjects and three readers were 4.15, 3.82, 3.35, and 3.2 for MEDI-SMV, TFIR, LN-QSM, and pTFI, respectively. Average clinical scores across three readers and the 17 hemorrhage patients were 3.95, 3.79, 2.89, and 3.19 for MEDI-SMV, TFIR, LN-QSM, and pTFI, respectively. The Fleiss' kappa interrater agreement was 0.36 with a standard error of 0.04, p < 0.05, and a [0.29 0.43] 95% confidence interval, constituting a reasonable agreement (Cohen, 1960).

### Filter Kernel Selection

**Figure 9:**
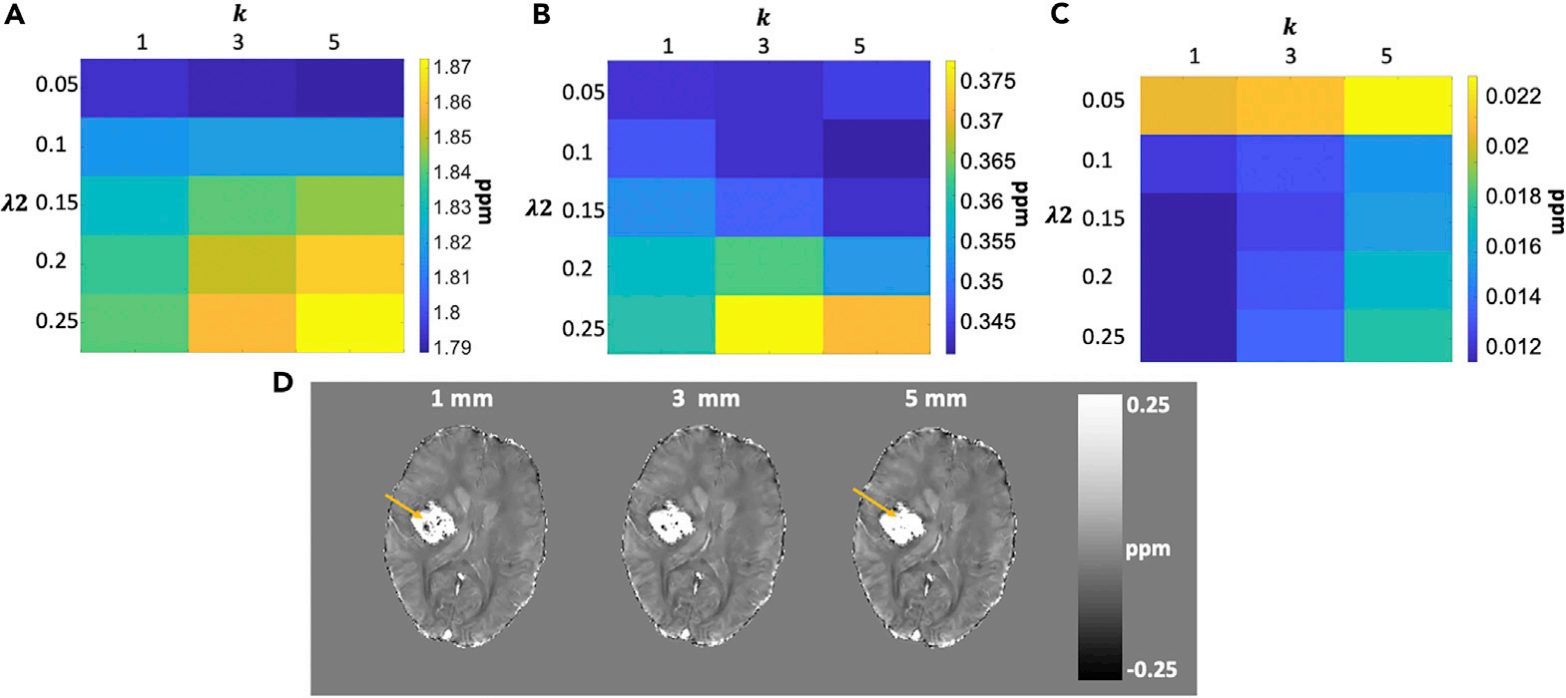
Filter Parameter Selection (A and B) (A) Effect of filter kernel on mean value of hemorrhage; (B) filter kernel effect on standard deviation of values within hemorrhage. (C and D) (C) Effect of filter kernel on the shadow index; (D) visible difference in high frequency content within hemorrhage using different kernel radii.

Figure 9A shows the hemorrhage mean value and standard deviation (normalized to the mean) for an average of nine subjects as a function of *λ*_2_ and *L* radius *k*. Using k = 5 mm leads to the largest mean hemorrhage susceptibility. A larger radius kernel suppresses variability and high frequency content within the hemorrhage for the optimal parameters. This is seen as a hemorrhage standard deviation (Figure 9B) and also in an example patient in Figure 9D. Figure 9C shows the shadow index as a function of *λ*_2_ and *k* averaged across nine subjects. Using a 1-mm kernel results in the lowest shadow index. This is also the radius obtained by minimizing reconstruction error using the COSMOS data as shown in Figure 2. As such, the shadow index and optimal error criterion are used to choose the 1-mm radius for *k* as optimal.

### Whole Head Mapping

**Figure 10:**
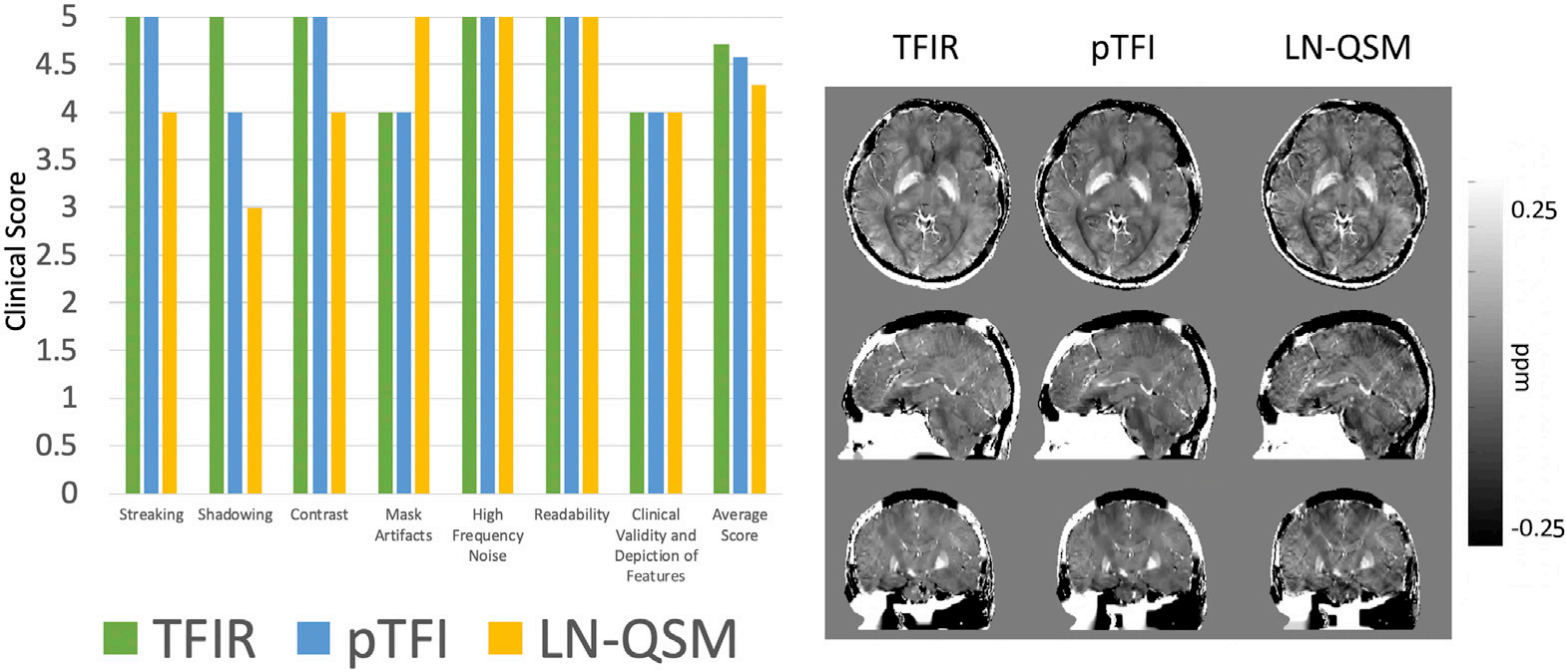
Whole Head Mapping Analysis Whole head masking with TFIR method (right) allows for total field reconstruction of the entire head. Clinical scoring (left) provides the comparison of methodologies.

Reconstruction of the susceptibility for the entire head, including the scalp, skull, and meninges is depicted in Figure 10. All total field methods are able to depict susceptibility across the field of view without substantial artifacts. The RMSE of the susceptibility within the brain (using the COSMOS reconstruction as ground truth) was 0.5846 for TFIR, 0.6003 for pTFI, and 0.7511 for LN-QSM. The average (across categories) clinical score was 4.71 for TFIR, 4.57 for pTFI, and 4.28 for LN-QSM. With regards to the skull region, LN-QSM showed susceptibility more consistent with that of bone compared with the other methods.

## Discussion

The data presented in this paper are strongly supportive of TFIR producing image quality that is close to being on par with the local field MEDI-SMV method, as compared with other total field methods. Both quantitative measures of shadow artifacts and clinical scoring presented here suggest that TFIR has fewer artifacts in the reconstruction and performs consistently across various datasets.

TFIR aims to reconstruct susceptibility utilizing signal intensity of the magnitude gradient data as a spatially adaptive regularization to solve the ill-posed optimization problem in QSM with fewer resultant artifacts, compared with the other methods investigated in this work.

Although this paper shows that TFIR improves the incidence of artifacts when compared with competing total field techniques, there is still residual shadowing artifacts in the TFIR method compared with MEDI-SMV. In future work, further automation of optimization parameters across a larger patient dataset is warranted. Incorporating other contrasts and information into the spatially adaptive masking term and spatial variation of the kernel size may further improve reconstruction quality. It is clear from Figure 6 that mid-brain and cerebellar nuclei are underestimated using total field methods. Because these regions have lower signal to noise ratio when observing the noise matrix and R2∗ map of the datasets, it seems that total field methods that do not utilize additional shadow suppression are prone to error in these regions. The utilization of denoising, contrast enhancement, incorporation of different MRI contrasts into the spatially adaptive mask, among other techniques, might result in further accuracy improvement. Various filters and transforms of the R2∗ map proposed in the literature could help correct for field inhomogeneities and susceptibility-derived artifacts (Walsh and Wilman, 2011; Yablonskiy et al., 2013). These will be explored in future research. A limitation of this study is the use of COSMOS as a ground truth susceptibility reconstruction. The latter may have compromised accuracy owing to local field extraction with multiple orientation datasets to reconstruct COSMOS itself. This could cause errors in the process of obtaining a ground truth map. This will be further investigated in future to provide more robust error measurements of various techniques. To further this, various other ROIs will be segmented and compared. It should be noted that, while the MEDI-SMV technique has excellent shadow-suppressive qualities owing to the use of the SMV low-frequency filter, it has a bias towards high-frequency fitting (Deh et al., 2015; Kee et al., 2017). Thus the ROI errors are notably higher for some of the high-susceptibility regions when compared with local field techniques without SMV filtration (Liu et al., 2017; Sun et al., 2018). This is an area for further exploration, and this points to the contrast enhancing effects of total field methods that may be applied to local field methods to further suppress shadows and obtain accurate fitting and contrast. Another limitation of this study is that the MEDI-SMV by design cannot be computed on the same mask as TFIR, LN-QSM, and pTFI, which do not necessitate erosion of the mask during dipole inversion. However, data (not shown) indicate that comparative effectiveness of shadow suppression by each method remains largely similar when TFIR, LN-QSM, and pTFI are reconstructed on the eroded mask used in MEDI-SMV, thereby not significantly altering the conclusions of this work.

A spatially variable *τ* and *L*, in addition to the use of a different type of filter entirely in place of *L*, might result is more optimal reconstructions. Furthermore, utilization of spatial information that better represents the regions outside of the brain and phase information corrected for chemical shift (Dong et al., 2015) will be important for whole head constructions, as shown in Figure 10 (Dong et al., 2015). Optimizing masking is another area of potential improvement, as it is known to affect image quality in both total and local field techniques. This will also be necessary for future automation of whole head reconstruction without skull stripping.

### Conclusion

This paper introduces an algorithm, TFIR, to perform susceptibility mapping from the total field using a spatially adaptive spatial frequency selective regularization term. This spatially adaptive regularization is based on a normalized signal magnitude at larger TEs given its correlation with susceptibility. Compared with other total field methods, TFIR showed increased accuracy in numerical and experimental phantoms and in COSMOS data and better quantitative and clinical scores in patient data. TFIR was nearly similar in quality to MEDI-SMV without the need for brain erosion.

### Limitations of the Study

As mentioned in the Discussion, there are several areas of expansion and limitation notable. For one, the local field method requires mask erosion; thus, it is challenging to determine the difference in accuracy and error within the eroded region in a comparative manner. Furthermore, COSMOS ground truth maps traditionally require local field extraction, leading to potential errors within the ground truth map generation. Variable filter selection of the TFIR low-pass L2 filter term will allow for further investigation of the shadow suppressive effect, which is an area for future expansion. Optimization of whole head mapping coupled with ground truth comparisons for the whole head will allow for total field methods to reach full clinical relevance.

### Resource Availability

#### Lead Contact

Further information and requests for data and/or code should be directed to and will be fulfilled by the Lead Contact, Pascal Spincemaille (pas2018@med.cornell.edu).

#### Materials Availability

This study did not generate new unique reagents.

#### Data and Code Availability

The code used for TFIR reconstructions is compatible with the Cornell MRI Research Lab. The TFIR code is a part of the QSM Toolbox, provided with full code and sample data, and located on the Cornell MRI Research webpage here http://pre.weill.cornell.edu/mri/pages/qsm.html.

There are restrictions to the availability of full sets of patient data owing to patient confidentiality measures. The following ethics statement provides further information on our data obtainment. All research conducted was reviewed by the Weill Cornell Medicine Institutional Review Board (Protocol Number 0909010639 and 1104011660). Whenever necessary, written informed consent was obtained by participants. Otherwise, data exemption 45 SFR 46.101(b) (4) is in place.

## Methods

All methods can be found in the accompanying Transparent Methods supplemental file.

## Supplemental Information

Document S1. Transparent Methods and Figures S1–S3

## References

[bib1] Acosta-Cabronero J., Williams G.B., Cardenas-Blanco A., Arnold R.J., Lupson V., Nestor P.J. (2013). In vivo quantitative susceptibility mapping (QSM) in Alzheimer’s disease. PLoS One.

[bib2] Chatnuntawech I., McDaniel P., Cauley S.F., Gagoski B.A., Langkammer C., Martin A., Grant P.E., Wald L.L., Setsompop K., Adalsteinsson E. (2017). Single-step quantitative susceptibility mapping with variational penalties. NMR Biomed..

[bib3] Cohen J. (1960). A coefficient of agreement for nominal scales. Educ. Physiol. Meas..

[bib4] Deh K., Nguyen T.D., Eskreis-Winkler S., Prince M.R., Spincemaille P., Gauthier S., Kovanlikaya I., Zhang Y., Wang Y. (2015). Reproducibility of quantitative susceptibility mapping in the brain at two field strengths from two vendors. J. Magn. Reson. Imaging.

[bib5] Deistung A., Schweser F., Wiestler B., Abello M., Roethke M., Sahm F., Wick W., Nagel A.M., Heiland S., Schlemmer H.P. (2013). Quantitative susceptibility mapping differentiates between blood depositions and calcifications in patients with glioblastoma. PLoS One.

[bib6] Dong J., Liu T., Chen F., Zhou D., Dimov A., Raj A., Cheng Q., Spincemaille P., Wang Y. (2015). Simultaneous phase unwrapping and removal of chemical shift (SPURS) using graph cuts: application in quantitative susceptibility mapping. IEEE Trans. Med. Imaging.

[bib7] Kee Y., Liu Z., Zhou L., Dimov A., Cho J., De Rochefort L., Seo J.K., Wang Y. (2017). Quantitative susceptibility mapping (QSM) algorithms: mathematical rationale and computational implementations. IEEE Trans. Biomed. Eng..

[bib8] Kirui D.K., Khalidov I., Wang Y., Batt C.A. (2013). Targeted near-IR hybrid magnetic nanoparticles for in vivo cancer therapy and imaging. Nanomedicine.

[bib9] Li X. (2011). Fine-granularity and spatially-adaptive regularization for projection-based image deblurring. IEEE Trans. Image Process..

[bib10] Li J., Lin H., Liu T., Zhang Z., Prince M.R., Gillen K., Yan X., Song Q., Hua T., Zhao X. (2018). Quantitative susceptibility mapping (QSM) minimizes interference from cellular pathology in R2∗ estimation of liver iron concentration. J. Magn. Reson. Imaging.

[bib11] Li W., Wang N., Yu F., Han H., Cao W., Romero R., Tantiwongkosi B., Duong T.Q., Liu C. (2015). A method for estimating and removing streaking artifacts in quantitative susceptibility mapping. Neuroimage.

[bib12] Liu T., Spincemaille P., De Rochefort L., Kressler B., Wang Y. (2009). Calculation of susceptibility through multiple orientation sampling (COSMOS): a method for conditioning the inverse problem from measured magnetic field map to susceptibility source image in MRI. Magn. Reson. Med..

[bib13] Liu T., Spincemaille P., de Rochefort L., Wong R., Prince M., Wang Y. (2010). Unambiguous identification of superparamagnetic iron oxide particles through quantitative susceptibility mapping of the nonlinear response to magnetic fields. Magn. Reson. Imaging.

[bib14] Liu T., Eskreis-Winkler S., Schweitzer A.D., Chen W., Kaplitt M.G., Tsiouris A.J., Wang Y. (2013). Improved subthalamic nucleus depiction with quantitative susceptibility mapping. Radiology.

[bib15] Liu T., Wisnieff C., Lou M., Chen W., Spincemaille P., Wang Y. (2013). Nonlinear formulation of the magnetic field to source relationship for robust quantitative susceptibility mapping. Magn. Reson. Med..

[bib16] Liu Z., Kee Y., Zhou D., Wang Y., Spincemaille P. (2017). Preconditioned total field inversion (TFI) method for quantitative susceptibility mapping. Magn. Reson. Med..

[bib17] Liu Z., Spincemaille P., Yao Y., Zhang Y., Wang Y. (2018). MEDI+0: Morphology enabled dipole inversion with automatic uniform cerebrospinal fluid zero reference for quantitative susceptibility mapping. Magn. Reson. Med..

[bib18] Liu Z., Wen Y., Spincemaille P., Zhang S., Yao Y., Nguyen T.D., Wang Y. (2020). Automated adaptive preconditioner for quantitative susceptibility mapping. Magn. Reson. Med..

[bib19] Murakami Y., Kakeda S., Watanabe K., Ueda I., Ogasawara A., Moriya J., Ide S., Futatsuya K., Sato T., Okada K. (2015). Usefulness of quantitative susceptibility mapping for the diagnosis of Parkinson disease. Am. J. Neuroradiol..

[bib20] Schweser F., Deistung A., Lehr B.W., Reichenbach J.R. (2011). Quantitative imaging of intrinsic magnetic tissue properties using MRI signal phase: an approach to in vivo brain iron metabolism?. Neuroimage.

[bib21] Sharma S.D., Hernando D., Horng D.E., Reeder S.B. (2005). A Joint Background Field Removal and Dipole Deconvolution Approach for Quantitative Susceptibility Mapping in the Liver.

[bib22] Shmueli K., De Zwart J.A., Van Gelderen P., Li T.Q., Dodd S.J., Duyn J.H. (2009). Magnetic susceptibility mapping of brain tissue in vivo using MRI phase data. Magn. Reson. Med..

[bib23] Song X., Xu Y., Dong F. (2015). A spatially adaptive total variation regularization method for electrical resistance tomography. Meas. Sci. Technol..

[bib24] Sun H., Ma Y., MacDonald M.E., Pike G.B. (2018). Whole head quantitative susceptibility mapping using a least-norm direct dipole inversion method. Neuroimage.

[bib25] Tan H., Liu T., Wu Y., Thacker J., Shenkar R., Mikati A.G., Shi C., Dykstra C., Wang Y., Prasad P.V. (2014). Evaluation of iron content in human cerebral cavernous malformation using quantitative susceptibility mapping. Invest. Radiol..

[bib26] Walsh A.J., Wilman A.H. (2011). Susceptibility phase imaging with comparison to R2 mapping of iron-rich deep grey matter. Neuroimage.

[bib27] Wang Y., Liu T. (2015). Quantitative susceptibility mapping (QSM): decoding MRI data for a tissue magnetic biomarker. Magn. Reson. Med..

[bib28] Yablonskiy D.A., Sukstanskii A.L., Luo J., Wang X. (2013). Voxel spread function method for correction of magnetic field inhomogeneity effects in quantitative gradient-echo-based MRI. Magn. Reson. Med..

