# Supplementary material for: Spatially Adaptive Regularization in Total Field Inversion for Quantitative Susceptibility Mapping
Source: iScience. 2020 Sep 12;23(10):101553. doi: 10.1016/j.isci.2020.101553 (PMC7522736; doi:10.1016/j.isci.2020.101553)
Supplement: Document S1. Transparent Methods and Figures S1–S3 [file mmc1.pdf]

iScience, Volume *23*

## Supplemental Information

# Spatially Adaptive Regularization

# in Total Field Inversion

# for Quantitative Susceptibility Mapping

**Priya S. Balasubramanian, Pascal Spincemaille, Lingfei Guo, Weiyuan Huang, Ilhami Kovanlikaya, and Yi Wang**

**SUPPLEMENTAL FIGURES**

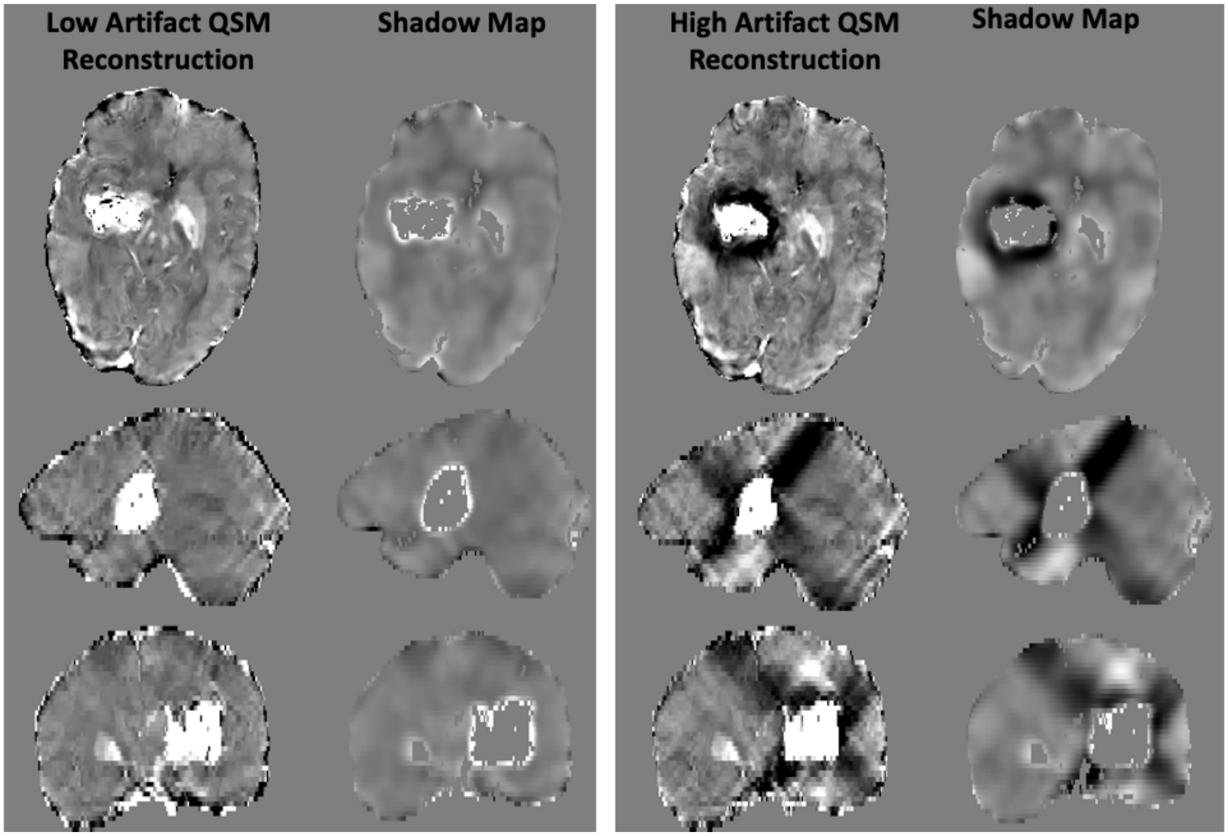


Figure S1. Shadow Map for Shadow Index, Related to Figures 7 and 8.

Shadow maps used in the calculation of the shadow index for the case of a low artifact (left) and high artifact (right) QSM reconstruction. The construction of the shadow map uses Equation T1 in the Transparent Methods. The shadow index is then the standard deviation within the shadow map.

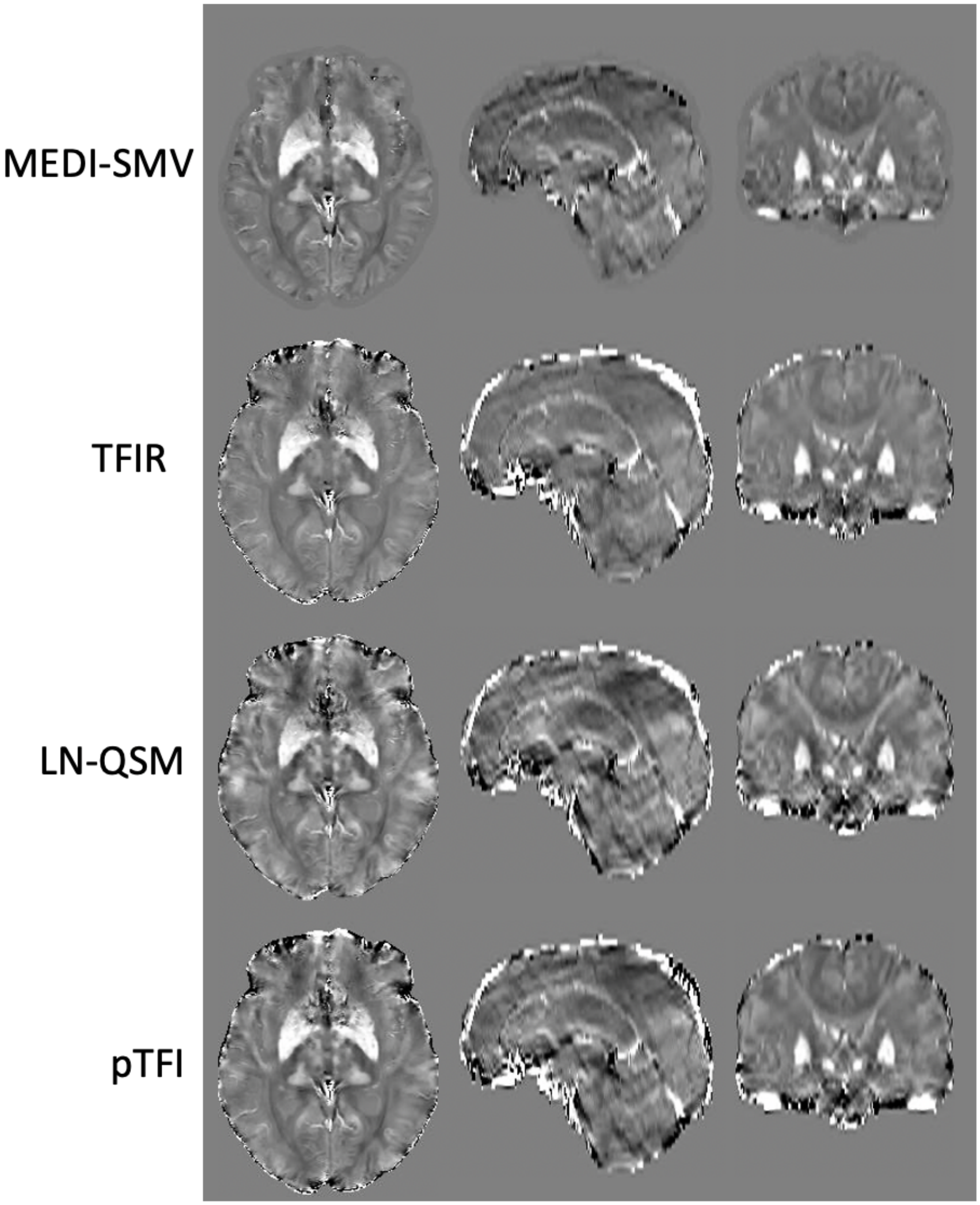


Figure S2. Non-Hemorrhage Case Comparison, Related to Figure 7.

Comparison of MEDI-SMV, LN-QSM, TFIR, and pTFI in a non-hemorrhage containing dataset. The pTFI, LN-QSM, and TFIR reconstructions use the original mask.

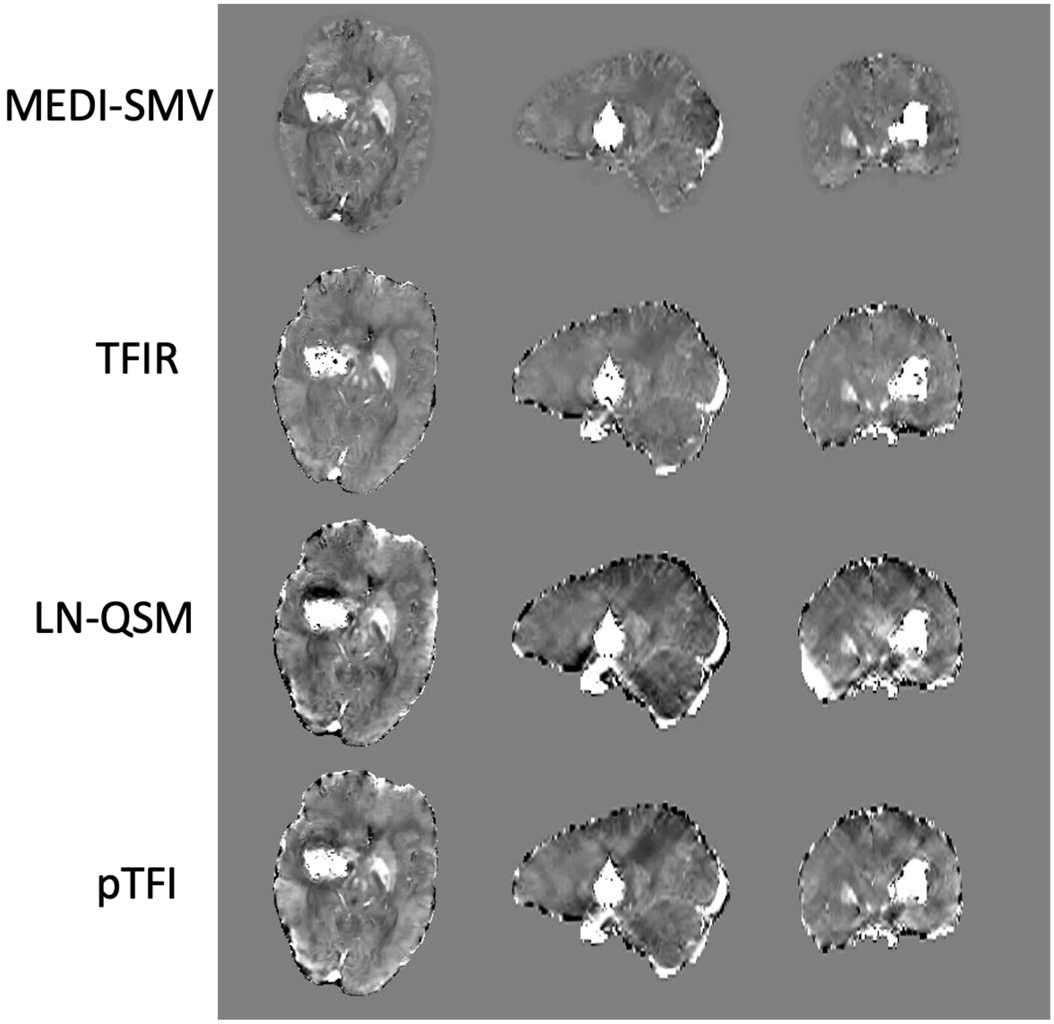


Figure S3. Hemorrhage Case Comparison, Related to Figure 8.

Comparison of MEDI-SMV, LN-QSM, TFIR, and pTFI in a non-hemorrhage containing dataset.  The pTFI, LN-QSM, and TFIR reconstructions use the original mask.

**TRANSPARENT METHODS**

Methods are reported in the below subsections, with computational and imaging parameters disclosed. The imaging parameters are all reported as native.

### 1. Gadolinium Phantom

An agarose gel matrix was used to create five wells (created by balloons) filled with Gadolinium solutions whose concentration ranged from 0.625 mM to 10 mM. Imaging was performed on a SIEMENS Prisma 3T scanner with a 32 channel head coil using a 3D multi-echo spoiled gradient recalled-echo (SPGR) sequence. Imaging parameters were: voxel size 0.6x0.6x0.8 $mm^3$, matrix size 256x256x106, 10 echoes with 4.1 ms echo spacing, TR / TE = 49 ms / 3.7 ms, 8 m 34 s scan time. $\lambda_1$ was set to $0.01$ and an edge mask based on a threshold such that 10% of voxels contained edges. Ground truth susceptibility values for the balloons were calculated using the Gd molar susceptibility of 0.33 ppm/mM.(De Rochefort *et al.*, 2010) The error was computed as $|QSM - \chi_{true}|/|\chi_{true}|$ per Gadolinium well and plotted on a per well basis. Regularization parameters for pTFI, MEDI-SMV, and LN-QSM were similarly selected to minimize error. The gadolinium phantom dataset is processed with total field for the total field reconstructions, with a numerically simulated 5 mm radius spherical 5 x $10^4$ ppm susceptibility source included to increase the background field present in the model. This source center is located at (-28, -28, 29) mm when taking the origin as the bottom right of the field of view shown in Figure 3.

### 2. Numerical Phantom

A numerical phantom was constructed based on the Zubal digital phantom. (Zubal, I George and Harrell, Charles R and Smith, Eileen O and Rattner, Zachary and Gindi, Gene and Hoffer, 1994) It contained a simulated hemorrhage with maximum susceptibility of 1 ppm. The R2* map was not low pass filtered. For error minimization and reconstruction, reconstruction was performed with 200 CG iterations with a 0.05 tolerance. The radius for the low pass operator $L$ was chosen as 1 mm radius through error minimization. Reconstruction parameters for TFIR, pTFI, MEDI-SMV, and LN-QSM were optimized by minimizing error with respect to the known ground truth. The numerical phantom is designed to have a substantial artificial background to mimic the brain and air interface, with the background set to a 9 ppm susceptibility.

## 3. Healthy subjects

Multiple orientation brain data was acquired in 4 healthy subjects. A COSMOS reconstruction provided the ground truth susceptibility map $\chi_{COSMOS}$.(Liu *et al.*, 2009) Example parameters include – imaging performed on a SIEMENS Prisma 3T scanner with a 32 channel head coil using a 3D multi-echo spoiled gradient recalled-echo (SPGR) sequence with the parameters being matrix size of 200x256x144 and voxel size of 1x1x1 $mm^3$ respectively, 6 echoes with 4.7 ms echo spacing, TR / TE = 26 ms / 4.7 ms, acquisition time 3 min 35 s, with partial Fourier, acceleration R = 2. Some of the healthy subject datasets are acquired on a 3T (GE, Waukesha, WI) MRI scanner. Example parameters include GE 32 channel head coil 3D multi-echo SPGR sequence with an acquired voxel size of 1x1x2 $mm^3$, acquisition matrix of 256x256x86, reconstructed matrix size of 512x512x172, TR / TE = 31.5 ms / 4.6 ms, acquisition time of 3 min 32 s. The above parameters are for one of the four datasets analyzed. For all methods, a reconstruction error was computed as $\sqrt{|\chi_{QSM} - \chi_{COSMOS}|/N}$, where the summation was over the COSMOS provided mask that erodes approximately 15 mm of the soft tissue mask. The default value $\lambda_1 = 0.001$ was used. Convergence criteria were kept at 150 CG iterations with 0.01 tolerance criteria. Reconstruction parameters for TFIR, pTFI, MEDI-SMV, and LN-QSM were optimized by minimizing the reconstruction error. For TFIR, the radius $k$ for the low-pass filter was included in the list of reconstruction parameters to be optimized over.

In addition to this analysis, the following regions of interest (ROI) were manually segmented by experienced radiologists on the COSMOS reconstruction of each subject: Globus Pallidus, Putamen, Caudate Nucleus, Red Nucleus, Dentate Nucleus, Substantia Nigra, Subthalamic Nucleus, and Thalamus. The average value within each ROI was recorded for all reconstruction methods (COSMOS, MEDI-SMV, TFIR, pTFI, and LN-QSM) and for all subjects.

## 4. Clinical data

Multiple echo gradient echo in N=33 consecutive patients imaged on two scanners (Siemens Skyra 3T and a Siemens Aera 1.5T) at our institution. Example imaging parameters included scanner with a 32 channel head coil using a 3D multi-echo spoiled gradient recalled-echo (SPGR) sequence with the parameters being voxel size 0.75-1.0 x 0.75-1.0 x 2.0-3.0 $mm^3$, 250-320 x 205-320 x 48-86 image size, 6 -10 echoes with 4.1-5.0 ms echo spacing, TR / TE = 39.8 – 43.2 ms /4.1-5 ms, imaging time 4 min 8-30 s. Some of the

scans were reconstructed to matrix sizes of 512x512x52-54 with resolutions of 0.5x0.5x3 $mm^3$. MEDI-SMV, TFIR, pTFI and LN-QSM were used to reconstruct susceptibility maps from these data. For TFIR, the kernel size $k$ for the low-pass filter $L$was set to that obtained in the COSMOS, which is 1 mm. The mask in which the MEDI-SMV result was available was used for computing quantitative measures (see below). Convergence criteria were kept at 150 CG iterations with 0.05 tolerance criteria.

***4a. Shadow Index***

Shadowing artifacts were quantified by a "shadow index" obtained as follows. First, starting from the brain mask $M$ (eroded by 5 mm to allow comparison with MEDI-SMV), all voxels whose susceptibility in absolute value was greater than 0.1 ppm were removed. The shadow mask is the region of interest in which the shadow map is defined. It is the region in which the susceptibility values are less than 0.1 ppm as shown in the extra figure. The mask itself is binary (0 outside, 1 inside) and excludes the hemorrhage and other susceptibility values greater than 0.1 ppm and the non brain portions. The shadow mask, $M_{shadowROI} = M \ \& \left|QSM_{ref}\right| < 0.1 \, ppm$ is used to define the region of the shadow map. Second, a "shadow map" was computed by multiplying the susceptibility map with this modified mask, followed by a 1 mm spherical mean convolution to filter out high spatial frequency components. The shadow map is the masked version of the filtered low frequency content. An expression may be given as

$$Shadow\ Map = E_{1mm}(\mathrm{M_{shadowROI}})\mathrm{SMV_{1mm}}(|\mathrm{QSM}|), \qquad \text{(T1)}$$

where $E_{1mm}$ is an erosion operation that removes a 1mm from the edge of a logical mask, $QSM_{ref}$ is the reference QSM that is used to removed high susceptibility magnitude regions, and SMV is the spherical mean operator, in this case with a 1mm radius.

Two examples of these shadow maps are shown in Supplemental Figure 1. From this map, the shadow index (in ppm) was computed by taking standard deviation of the map within the mask. Supplemental Figure 1 shows a low (left) and a high (right) shadow artifact example. This shadow index thus takes into account artifacts rather than properly fitted high susceptibility regions or high frequency morphological information of the brain. This method is applied to both hemorrhage and non-hemorrhage datasets. Given the large magnitude susceptibility within the hemorrhage, the hemorrhage region is excluded, along with other >0.1ppm susceptibilities, from the shadowing calculation.

***4b. Clinical Scoring***

Image scoring was performed by 3 experienced radiologists (WH (13 years experience), LG (7 years experience), IK (32 years experience)). The readers were asked to score each of the following categories 1) streaking, 2) shadowing 3) image contrast, 4) mask erosion and mask related artifacts, 5) high frequency noise (checkerboard and ripple artifacts), 6) overall readability, 7) clinical validity and realistic depiction of features. An additional score was computed by taking the mean over these 7 categories. For each category, the reader was asked to assign a score between 1 and 5. A score of 1 corresponds to many artifacts and the lowest image quality. A score of 5 corresponds to the highest image quality and fewest artifacts. The scores for all three readers were averaged per scan for a datasets analyzed in the quantitative analysis section (16 non-hemorrhage and 17 hemorrhage). This is also completed for the whole head reconstruction (1 case of healthy subject). Interrater repeatability was assessed using Fleiss' kappa.

### *4c. Hemorrhage Analysis*

The hemorrhage intensities are analyzed across different reconstructions by thresholding > 0.2 ppm to extract the hemorrhage region. This region is then averaged, and the mean and standard deviation is presented for the N=17 cases.

For the hemorrhage in vivo analysis, and the hemorrhage containing numerical phantom, LN-QSM was modified with modifications suggested in the original work in the whole head reconstruction section. An L2 regularization specific mask was constructed by removing from the brain mask those voxels whose magnitude fell below 25% of the maximum magnitude signal within the field of view, designated as $M_{mag}$ below. These changes followed similar modifications proposed in Sun et. al for whole brain susceptibility mapping, except that, in our work, changes in the mask were only made for the L2 regularization term and not in the data fidelity and L1 term. The modified cost function for LN-QSM was then.

$$\chi^* = \underset{\chi}{\operatorname{argmin}} \frac{1}{2} \|w(f - d * \chi)\|_2^2 + \lambda_1 \|M_G \nabla \chi\|_1 + \lambda_2 \left\|M_{mag} \chi\right\|_2^2 \quad \text{(T2)}$$

Optimization of the regularization parameters $\lambda_1$and $\lambda_2$were obtained by minimizing the error with respect to the ground truth (COSMOS) reconstruction, as was done for all methods.

### 5. Kernel Evaluation and Selection

To evaluate the influence of the choice of kernel on the TFIR image quality, the radius $k$ of the kernel for the low pass filter $L$and $\lambda_2$were changed over a number of values (1mm, 3mm, 5mm and 0.05-0.25 across

each radius). It is hypothesized that the size of the kernel will influence the contrast and accuracy of the resultant susceptibility map, as the filter kernel size effectively selects different spatial frequency as it is varied. As such, various quantities are analyzed for the range of parameters selected. For each radius, contrast and artifacts were quantified in subjects with hemorrhage by quantifying the mean value of the hemorrhage region, the standard deviation of the hemorrhage region, and the shadow index outside the hemorrhage ROI (as a measure of shadow artifact). This analysis was performed in nine hemorrhage in vivo, clinical datasets.

### 6. Whole Head Mapping

TFIR was used to map the susceptibility for all soft tissue within the field of view. The dataset utilized was one of the COSMOS datasets (N=1, healthy subject). A soft tissue mask was constructed by thresholding the magnitude of the first echo. Imaging was performed on a 3T (GE, Waukesha, WI) MRI scanner with a 32 channel head coil using a 3D multi-echo spoiled gradient recalled-echo (SPGR) sequence with the parameters being voxel size 1x1x2 $mm^3$, 256x256x86 image size, 6 echoes with 2.7 ms echo spacing, TR / TE = 26 ms / 2.7 ms.
